# Trajectories of Metabolic Syndrome Development in Young Adults

**DOI:** 10.1371/journal.pone.0111647

**Published:** 2014-11-04

**Authors:** Vivian T. W. Poon, Jennifer L. Kuk, Chris I. Ardern

**Affiliations:** School of Kinesiology and Health Science, York University, Toronto, ON, Canada; University of the Balearic Islands, Spain

## Abstract

**Background:**

Metabolic syndrome (MetS) is a constellation of metabolic aberrations that collectively increase the risk for cardiovascular disease and type 2 diabetes. Greater understanding of MetS developments may provide insight into targeted prevention strategies for individuals at greatest risk. The purpose of this study was to i) identify distinct patterns of longitudinal MetS development and; ii) develop a character profile that differentiates groups by level of MetS risk.

**Methods and Results:**

Data from the Coronary Artery Risk Development in Young Adults (CARDIA) study (n = 3 804; 18–30 y) was obtained by limited access application from the National Heart, Lung, and Blood Institute and used for this analysis. MetS, as defined by the Harmonized criteria, was assessed over a 20 year follow-up period. Group-level trajectory analysis identified 4 distinct groups with varying rates of component development [No (23.8% of sample); Low (33.5%); Moderate (35.3%); and High MetS (7.4%)]. After adjusting for covariates, individuals in the At-Risk groups (Low, Moderate and High MetS) were more likely to be of black ethnicity (1.37, 1.14–1.66), have a family history of cardiovascular disease (1.61, 1.31–1.97) and history of dieting (1.69, 1.20–2.39) when compared to the No Risk trajectory group (No MetS). Conversely, increasing baseline education (0.76, 0.65–0.89) and aerobic fitness (0.55, 0.47–0.64) was inversely associated with At-Risk group membership.

**Conclusions:**

Results suggest distinct profiles of MetS development that can be identified by baseline risk factors. Further research is necessary to understand the clinical implication of intermediate MetS development groups with respect to overall cardiometabolic risk.

## Introduction

Metabolic syndrome (MetS) is a constellation of cardiovascular risk factors that collectively increases the risk and burden of chronic disease, particularly type 2 diabetes [1] and cardiovascular disease (CVD) [2]. Strategies to detect those at high-risk of cardiometabolic diseases at a pre-clinical stage are therefore necessary. Although it is now well-established that cardiovascular risk tends to track from childhood into adulthood [3], [4], most efforts to address overt risk do not begin until early adulthood [5]. Moreover, the paths of MetS development and contributing risk factors have been left largely undescribed.

Previous research into the longitudinal development of MetS components and their medical sequalae has been hampered by two distinct limitations. First, research into the development of MetS has been focused primarily on the five individual MetS components (hypertension, hypertriglyceridemia, low high-density lipoprotein-cholesterol (HDL-C), elevated waist circumference (WC) and high fasting plasma glucose), with little evaluation of both the overall pattern of development, nor their association with known primordial risk factors such as gender, ethnicity, physical inactivity, and diet. Secondly, conventional approaches to exploring patterns of MetS development are typically based on parametric assumptions, whereby it has been assumed that all individuals with MetS develop the condition in a similar manner. Such efforts have subsequently led to strategic, yet arbitrary categorizations of study samples that may not reflect the full range of variation in MetS component combinations possible [6]. The aims of this current study were to therefore: 1) identify distinct patterns of longitudinal MetS development, and; 2) evaluate how baseline non-modifiable (gender, ethnicity and family history) and modifiable (physical activity, aerobic fitness, dietary practice, education, smoking, and alcohol consumption) risk factors may contribute to MetS trajectories in young adults.

## Research Design and Methods

### Data Access and Ethics Statement

Data for this analysis were obtained through a limited access dataset obtained from the National Heart, Lung, and Blood Institute of the National Institutes of Health. The CARDIA study was approved by institutional review boards at all study locations. This research was reviewed and approved by the Human Participants Review Sub-Committee of York University. Written Informed Consent was provided by all CARDIA study participants.

### Coronary Artery Disease Risk Development in Young Adults (CARDIA)

The Coronary Artery Disease Risk Development in Young Adults (CARDIA) study is an ongoing, multicenter, longitudinal study designed to track the development of CVD risk factors in a U.S. cohort of apparently healthy 18–30 year old participants. Details of the scientific rationale, eligibility requirements, recruitment process and baseline characteristics of the CARDIA participants have been published elsewhere [7]. In 1985–1986, participants were randomly sampled from Birmingham, AL; Chicago, IL; Minneapolis, MN and from the Kaiser-Permanente health plan in Oakland, CA. Overall, 5 115 men and women were recruited and balanced based on gender, ethnicity (black and white), education (≤ high school graduate or > high school graduate), and age (18–24 years old or 25–30 years old). Six clinical examinations were subsequently performed: baseline (1985–1986), and 2, 5, 7, 10, 15 and 20 years follow-up, with participant retention of 90%, 86%, 81%, 79%, 74% and 72%, respectively. Since fasting plasma glucose measurements were not collected at year 2 or year 5, both collection dates were omitted from the current analysis.

The procedures for each examination differed marginally, as efforts were made to ensure that emerging cardiovascular risk factors were captured. However, each examination consistently included physical measurements (e.g. blood serum chemistry, blood pressure, and anthropometry), lifestyle factors (e.g. physical activity (PA), dietary habits, tobacco and alcohol use, behavioural/psychological assessment), a medical history and socioeconomic status (SES) profile.

### Study Inclusions and Exclusions

Data was limited to participants who attended at least two follow-up examinations, were non-pregnant, free from MetS at baseline, and had complete data for all five MetS components. Of the initial 5 115 individuals enrolled at baseline, 3 804 apparently healthy participants completed at least two follow-up examinations (males: n = 1 694; females: n = 2 110). Collectively, 2 597 attended all five examinations, while 718 completed only four visits and 489 attended only three.

### Dependent Variables

All outcome variables were collected according to a standardized protocol and processed at central laboratories. Participants were asked to fast for at least 12-hours and refrain from engaging in any heavy physical activity or tobacco use in the 2-hours prior to testing [7]. Plasma triglycerides (TG) were measured using enzymatic procedures, and high density lipoprotein cholesterol (HDL-C) was measured after dextran sulphate-magnesium precipitation. Serum low density lipoprotein cholesterol (LDL-C) was estimated based on the Friedewald equation [LDL-C = total cholesterol - HDL-C - (TG/5)], where TG<4.52 mmol/L [8]. Blood pressure was measured using a Hawksley random-zero sphygmomanometer after 5 minutes of seated rest. Blood pressure was measured three times with 1-minute intervals, with mean values of the last two systolic (SBP) and diastolic (DBP) measures being used for analysis. All anthropometric measurements were assessed on two occasions under the supervision of two lab technicians. Waist circumference (WC) was measured at the minimum waist girth (to the nearest 0.5 cm) [7]. This process was consistent across all examinations and study centers.

For the purpose of the current analysis, MetS was operationalized by the Harmonized criteria [9], defined as three or more of abdominal obesity (male: waist circumference: ≥102 cm; female: ≥88 cm), high blood pressure (systolic ≥130 mmHg or diastolic ≥85 mmHg or drug treatment for hypertension), high triglyceride levels (≥1.7 mmol/L (mM) or drug treatment for elevated triglyceride or cholesterol), low high density lipoprotein cholesterol (HDL-C; men: <1.0 mM; women: <1.3 mM), and high fasting plasma glucose (≥5.6 mM or diagnosed diabetes)] at each examination.

To provide further insight into the relationship between MetS and global cardiovascular risk, the broader framework of the revised *Third Report of National Cholesterol Education Program Expert Panel on Detection, Evaluation, and Treatment of High Blood Cholesterol in Adults* (ATP III-R) [10] was used. In the first step, traditional CHD risk factors (smoking, hypertension, low HDL-C, family history of CHD, and age) were counted. If participants were observed to have two or more risk factors, the second step involved calculating 10-year CHD risk (based on the Framingham Risk Algorithm [11]), and the presence of pre-existing CHD or CHD risk equivalents (previous CHD, diabetes, and ≥20% 10-year risk of CHD). Participants were ultimately placed into one of the four ATP III-R risk groups (High, Moderately-High, Moderate, and Lower Risk) and assigned to a treatment category (No Treatment, Therapeutic Lifestyle Change (TLC; dietary modification, weight management, and physical activity), or Drug Eligible) on the basis of their global risk status and personalized LDL-C goal.

### Independent Variables

Covariates included both non-modifiable (sex, ethnicity and family history of CHD) and modifiable risk factors (physical activity, cardiorespiratory fitness, diet, smoking, alcohol consumption, and education). All non-modifiable risk factors were collected by self-report using standardized questionnaires.

Because half of the selected participants were between the ages of 18 and 24 years at baseline, self-reported highest level of education (less than high school education, high school graduate, college or university degree, and postgraduate studies) was re-assessed at each follow-up interval.

Baseline PA was based on an interview-administered questionnaire derived from the Minnesota Leisure Time Physical Activity Questionnaire. Subsequently, the CARDIA Physical Activity Questionnaire provides an estimate of 12-month PA frequency based on participation in 13 activities. Because participants were not asked to report the duration of PA, separate heavy and moderate intensity activity scores (expressed in exercise units; EU) were computed based on the product of the frequency of activity participation and activity intensity [12]. A total PA score was derived by summing the moderate and heavy sub-scores. While the CARDIA PA scores cannot be directly compared against caloric expenditures, it has been previously reported that 200 EU are approximately equivalent to regularly engaging in exercise at 6 METS (i.e. stationary bicycling or swimming), 2 hours/week for 11 months per year [13]. For ease of comparison across groups, continuous scores were categorized into sex-specific tertiles (“Inactive”, “Moderately Active”, and “Active”).

Aerobic fitness was estimated by the modified Balke treadmill test [12]. Participants with a history of CHD, CVD medications, physician assessed chronic conditions, or abnormal pulmonary function were exempt from the fitness testing protocols (n = 48). Consistent with previous research, participants in the lowest sex-specific quintile were classified as “unfit”, the 20^th^ to 60^th^ percentile were classified as “moderately fit”, and those exceeding the 60^th^ percentile were deemed “high fit” [14].

Usual alcohol consumption was estimated using the CARDIA Alcohol Use Questionnaire, which asks participants to report their typical weekly consumption of wine/beer/liquor over the past year. Consistent with Center for Disease Control (CDC) guidelines, a standard alcoholic drink (with 13.7 g of pure alcohol) [15] was used to establish the average number of drinks per day and used to categorize participants as non-drinkers (no drinks/day), moderate drinkers (1–2 drinks/day), and heavy drinkers (≥3 drinks/day) [14]. Smoking status (current, former, and never smokers) was assessed using the Tobacco Questionnaire and Follow-up Questionnaire, and fast food consumption was self-reported as frequency per week or month and subsequently converted to reflect weekly intake. Finally, participants were asked to indicate whether they were currently engaged in efforts to reduce their weight through dietary restriction (yes/no).

### Statistical Analysis

Trajectory modelling [16], a semi-parametric group-based mixed model, was used to explore the development of MetS and its individual components. By using a customized SAS macro (PROC TRAJ) [17] it was possible to explore multiple distinct pathways or patterns in MetS development while simultaneously accounting for unobserved heterogeneity in the data. Data analysis proceeded through three steps. First, MetS development trajectories were determined using PROC TRAJ. All trajectories were modeled as a function of age to identify the probability of developing MetS as well the number of MetS components. Consistent with previous literature [16], [18], [19], the Bayesian Information Criterion (BIC), posterior probabilities, and descriptive statistics were used to evaluate the fit of the most parsimonious model. Briefly, the best model is the one with the smallest negative BIC (in the most complex model). When a model is well-defined, each study participant will have a high probability of belonging to one particular trajectory, and a low probability of belonging to the others. The calculated trajectory then represents the probability of group membership over time.

Once all participants had been assigned to a trajectory group, group-level profiles (e.g. modifiable and non-modifiable characteristics) were evaluated by Chi-square and analysis of variance, as appropriate. To assess potential baseline predictors of MetS, the pattern of individual MetS components was plotted separately for each group across the 20-years of follow-up. Additionally, measures of metabolic and cardiovascular risk (MetS development, Framingham Risk, ATP III-R risk and treatment groups) were assessed both at baseline and compared by MetS group at year 20.

To identify factors associated with higher risk group membership, groups with similar risk profiles and trajectories were then aggregated into either “no risk” or “at-risk” groups. Logistic regression was then used to identify variables that could predict group membership and identify targets for lifestyle-based intervention. Model 1 adjusted for non-modifiable risk factors (i.e. sex, ethnicity, and family history of CVD), and Model 2 additionally adjusted for modifiable and behavioural risk factors (i.e. additional adjustment for education, smoking, drinking, physical activity score, current engagement in weight reducing diets, and fast food consumption). After assessing collinearity and confirming independence (Tolerance<0.25; VIF<4), a further model adjusted for both non-modifiable and modifiable risk factors as well as aerobic fitness (Model 3). All statistical analyses were conducted using SAS version 9.3 (Cary, NC, U.S.A.) with statistical significance set at alpha <0.05.

## Results

### Identification of Trajectory Groups

**Figure 1:**
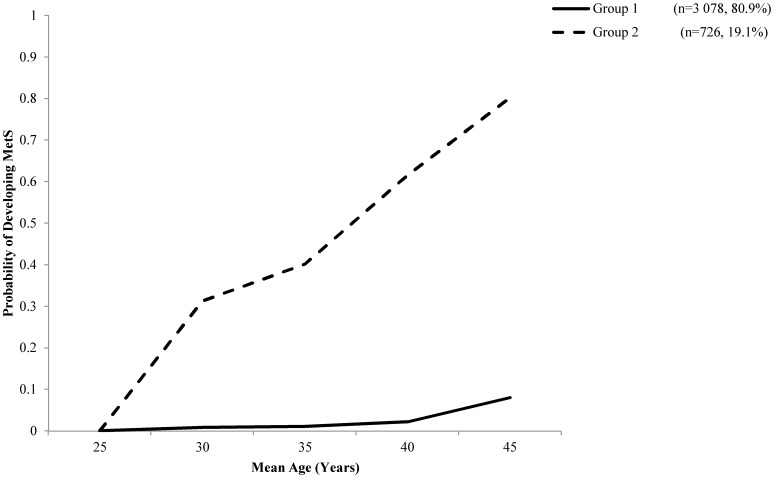
Group-based trajectory of MetS development probability over a 20-year span as determined by PROC TRAJ logit modelling in 3 804 apparently healthy young adults. Two distinct groups were observed and was denoted as Low Probability (n = 3 078) and High Probability (n = 746).

The 20-year likelihood of developing MetS was found to follow two distinct trajectories. Group 1 (MetS: n = 3 078, 80.9% of the sample) demonstrated a steady low-probability of developing MetS, while group 2 (MetS: n = 726, 19.1%) increased linearly over time, reaching approximately 80% likelihood by the final year (Figure 1).

**Figure 2:**
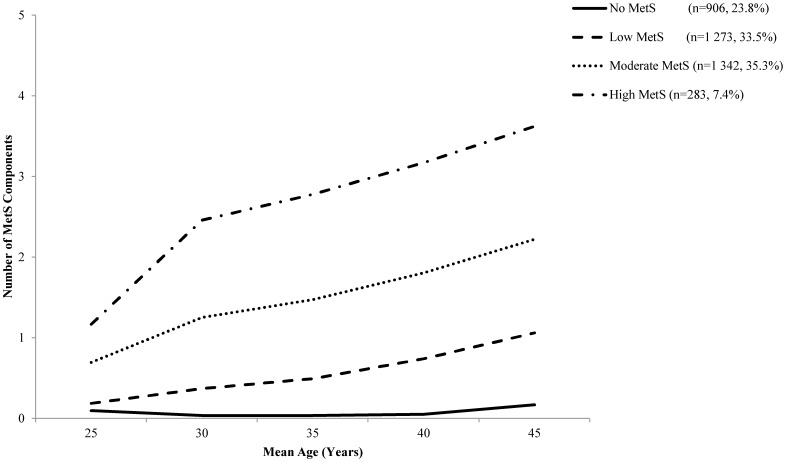
Group-based trajectory of MetS development based on the number of components attained over a 20-year span. Trajectories were determined by PROC TRAJ censored normal modelling in 3 804 apparently healthy young adults. Four trajectories were observed and denoted as No MetS (No MetS: n = 906), Low MetS (n = 1 273), Moderate MetS (n = 1 342), and High MetS (High MetS: n = 283).

When trajectory models were developed with the number of MetS *components* as the outcome, a four group model was found to be the best fit model (Figure 2) and was selected based on the observed BIC statistics (Table S1), posterior probabilities (Table S2), and descriptive results (Table 1). Briefly, The final model therefore included: i) a group of individuals with nearly no increase in the number of MetS components (No MetS: n = 906, 23.8% of sample); ii) a group that demonstrated slow development of MetS components (Low MetS: n = 1 273, 33.5% of sample); iii) a group with a linear increase up to two MetS components (Moderate MetS: n = 1 342, 35.3%), and; iv) a group with pre-existing MetS components followed by rapid development of additional MetS components (High MetS: n = 283, 7.4%). While the observed trajectory patterns represent that of the latent (estimated) development of MetS components, they should not be considered actual categories of growth.

**Table 1:** Characteristics at Baseline and Year 20§ by Trajectory Groups.

	Trajectory Groups							
	No		Low		Moderate		High	
	Baseline	Year 20	Baseline	Year 20	Baseline	Year 20	Baseline	Year 20
	*n = 906*	*n = 770*	*n = 1 273*	*n = 1 094*	*n = 1 342*	*n = 1 065*	*n = 283*	*n = 215*
Non-Modifiable Risk Factors								
Sex (% Male)† ‡	41.5	39.7	49.0	47.4	42.1	41.3	45.6	44.7
Ethnicity (% Black) † ‡	36.8	33.1	47.8	45.2	57	55.1	58.3	59.1
Family History of CHD (%) †	19.7		28.3		30.9		36.4	
Modifiable Risk Factors								
Education (% ≤ High School Graduates) † ‡	4.8	28.6*	6.8	36.5*	9.0	46.9*	9.7	50.0*
Smoking (% Current) † ‡	22.0	13.9*	27.2	17.1*	32.1	23.8*	30.9	19.7*
Alcohol (% Non-drinker) † ‡	71.5	70.1	70.1	70.2	72.5	78.3*	80.5	84.9
Physical Activity Level (% Inactive) †	27.7	34.8*	30.3	39.7*	35.5	52.1*	37.5	60.8*
Aerobic Fitness (% Unfit) † ‡	11.0	9.8*	8.9	24.3*	22.5	50.1*	38.9	71.3*
Fast Food Consumption (% more than 2 times/week) † ‡	25.7	17.0*	28.0	23.8*	33.4	26.0*	30.0	30.4
Currently on a Weight Reducing Diet (%) † ‡	5.6	25.9*	7.8	33.2*	9.7	35.5*	13.6	38.0*
MetS Components								
Systolic Blood Pressure (mmHg) † ‡	106.6±9.4	109.3±11.1*	109.5±9.8	116.4±13.7*	111.7±11.5	119.9±16.7*	115.1±10.5	124.9±16.5*
Diastolic Blood Pressure(mmHg) † ‡	66.5±8.6	66.6±8.2	68.0±9.0	72.2±10.5*	69.1±10.0	76.6±11.9*	72.1±9.9	81.4±11.1*
Waist Circumference (cm) *Males* † ‡	77.5±5.3	87.1±7.4*	79.3±6.9	93.0±9.6*	83.5±8.6	101.4±12.8*	91.1±9.2	114.4±12.2*
Waist Circumference (cm) *Females* † ‡	67.8±5.4	75.3±6.9*	70.1±6.3	83.9±10.7*	78.0±11.4	95.8±13.1*	87.4±12.4	107.9±11.5*
Fasting Plasma Glucose mmol/L) † ‡	4.4±0.4	4.9±0.4*	4.5±0.5	5.2±0.8*	4.6±0.8	5.6±1.4*	4.8±0.7	6.9±2.9*
Serum Triglycerides (mmol/L) † ‡	0.6±0.3	0.8±0.3*	0.7±0.4	1.0±0.6*	0.9±0.5	1.5±1.0*	1.2±0.6	2.1±1.3*
HDL-C (mmol/L) *- Males* † ‡	1.5±0.1	1.5±0.2	1.4±0.1	1.3±0.2	1.1±0.1	0.9±0.1	1.1±0.1	1.0±0.1
HDL-C (mmol/L) *- Females* † ‡	1.6±0.1	1.9±0.2	1.5±0.1	1.6±0.1	1.3±0.1	1.3±0.2	1.1±0.1	1.1±0.1
Health Risk								
Obesity (% Body Mass Index ≥30 kg/m^2^) † ‡	0.3	5.4*	2.3	23.6*	17.3	45.7*	42.4*	66.1*
Develop Metabolic Syndrome (%) ‡	–	0*	–	5.6*	–	40.5*	–	93.0*
No MetS Components (%) † ‡	91.1	86.1*	83.7	27.1*	41.3	3.3*	19.2	0.0*
Framingham Risk Score	1.0±0.2	1.8±2.0*	1.1±0.4	2.2±2.6*	1.1±0.5	2.5±3.2*	1.1±0.6	2.5±3.4*
ATP-III-R Group (% High Risk) ‡	0	0	0.5	3.2*	0.2	10.8*	0.1	33.5*
ATP-III-R Treatment (% Drug Therapy) † ‡	0.2	0.8	0.5	3.4*	1.5	8.5*	0.4	24.9*

Data presented as group means ± SD unless stated otherwise;

*Significance observed between baseline and Year 20, within each trajectory group; p<0.05;

† Significance observed between trajectory groups at baseline; p<0.05;

‡ Significance observed between trajectory groups at Year 20; p<0.05;

§ Presented Year 20 data reflects those who attended that specific exam; Abbreviations: CHD; Coronary Heart Disease, HDL-C; High Density Lipoprotein Cholesterol; MetS, Metabolic Syndrome; ATP III-R, Revised Third Report of National Cholesterol Education Program Expert Panel on Detection, Evaluation, and Treatment of High Blood Cholesterol.

**Figure 3:**
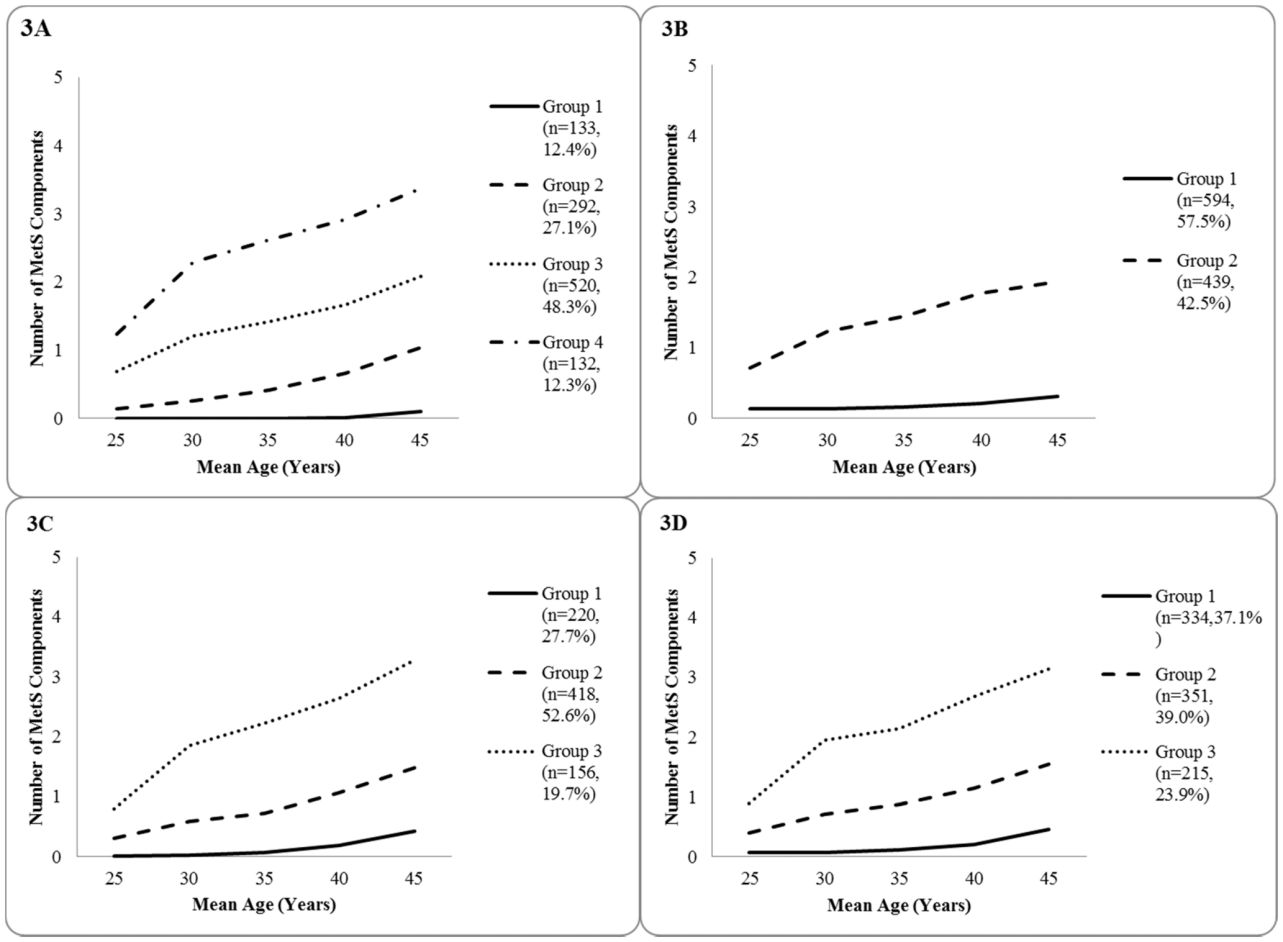
Ethnic-gender specific group-based trajectory of MetS development probability over a 20-year span as determined by PROC TRAJ censored normal modelling in 3 804 apparently healthy young adults. Figure 3A depicts four distinct trajectories among Black females (n = 1 077). Figure 3B depicts two distinct trajectories among White females (n = 1 033). Figure 3C depicts three distinct trajectories among Black males (n = 794). Figure 3D depicts three distinct trajectories among Black males (n = 900).

Potential gender and ethnic differences within the cohort were also assessed, and gender- and ethnic-specific models were developed (Figure 3). Analyses yielded four distinct trajectories among black women, and two groups among white women. Amongst male participants, both black and white cohorts presented similar trajectory plots with three distinct groups each. Due to sample size constraints, extensive post-hoc analyses could not be conducted, meaning that predictive modeling was limited to the overall pooled trajectories (Figure 2).

### Evolution of Characteristics and MetS Components within Trajectory Groups

**Figure 4:**
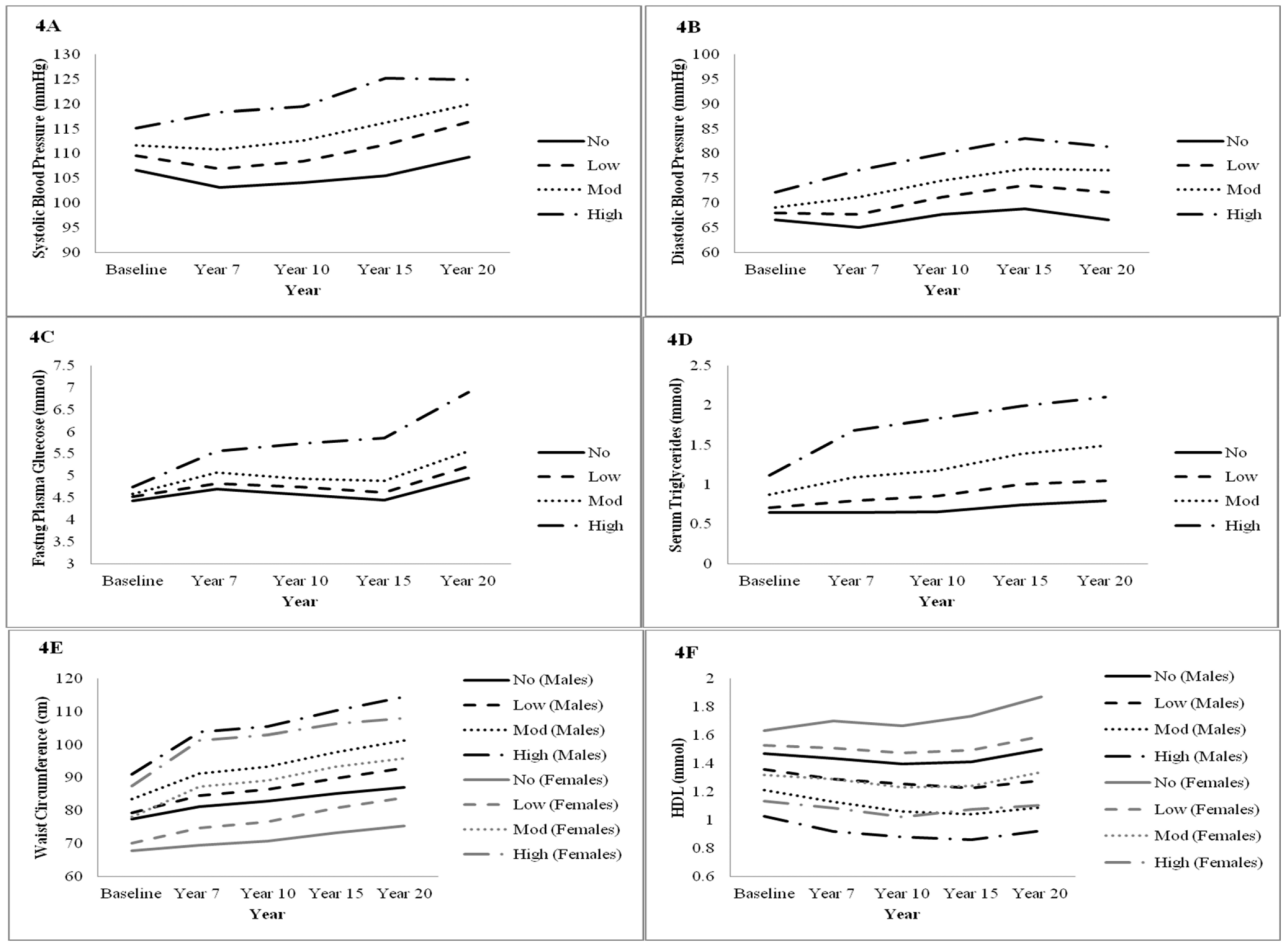
Evolution of individual MetS Components based on trajectory groups (No, Low, Mod and High).

Baseline characteristics between each of the trajectory groups are presented in Table 1. Compared to the No MetS group, High MetS group members were more likely to be non-drinkers, black ethnicity, obese, consume fast food more than twice a week, and have lower PA, aerobic fitness level, and family history of CHD. Differences in biophysical characteristics were also observed (Table 1 and Figure 4). Most notably, the mean WC was 13.6 cm larger amongst males and 19.7 cm amongst females in the High MetS group than the No MetS group. Similarly, compared to the No MetS group, both TG and HDL levels were 0.5 mM higher in the High MetS group.

Mean differences in the MetS components observed at baseline persisted through year 20, wherein the High MetS group presented significantly higher levels of all five MetS components (Table 1 and Figure 4). Indeed, at year 20 both Moderate and High MetS trajectory groups had mean fasting plasma glucose levels that exceed that of MetS defined cut-offs, with marked increases at the final follow-up assessment. Across follow-up, average WC increased in all trajectory groups, but was most pronounced for the Moderate and High MetS group. Substantial increases in the prevalence of obesity were also observed, particularly amongst Moderate (Baseline: 17.3% vs. Year 20: 45.7%) and High MetS (Baseline: 42.4% vs. Year 20: 66.1%) groups.

Consistent with estimated trajectory patterns, higher risk trajectory groups corresponded to a growing prevalence of MetS by year 20 (No: 0%; Low: 5.6%; Moderate: 40.5%; High: 93.0%). However, the short-term risk of CVD (as predicted by the Framingham Risk Algorithm) among CARDIA participants at baseline was low (Baseline: 1.0 to 1.1% 10-year risk vs. Year 20: 1.8 to 2.5% 10-year risk). By contrast, group differences were observed when ATP III-R global risk strata were applied. In general, increasing risk trajectory corresponded with greater cardiometabolic risk, with 25% of all High MetS members eligible for lipid-lowering therapy by year 20.

To identify baseline predictors associated with membership in at-risk trajectory groups, logistic regression was used to compare at-risk trajectory groups (Low, Moderate and High MetS) to the apparent no risk (No MetS) group (Table 2).

**Table 2:** Baseline Predictors of Higher Trajectory Group Membership.*

	Model 1	Model 2	Model 3
Group Predictors†	Odds Ratio	Odds Ratio	Odds Ratio
	(95% CI)	(95% CI)	(95% CI)
No vs At-Risk	*n = 2 898*	*n = 2 528*	*n = 2 467*
Gender (Female vs Male)	0.81 (0.70–0.95)	0.85 (0.71–1.01)	0.86 (0.72–1.04)
Ethnicity (Black vs White)	1.93 (1.65–2.25)	1.66 (1.38–1.98)	1.37 (1.14–1.66)
Family History of CVD (yes vs no)	1.74 (1.44–2.09)	1.71 (1.40–2.09)	1.61 (1.31–1.97)
Education	-	0.72 (0.61–0.83)	0.76 (0.65–0.89)
Smoking	-	1.10 (0.99–1.21)	1.06 (0.96–1.18)
Drinking	-	1.00 (0.85–1.16)	0.99 (0.84–1.16)
PA Level	-	0.88 (0.79–0.98)	0.98 (0.88–1.09)
Currently Dieting	-	1.92 (1.37–2.70)	1.69 (1.20–2.39)
Fast Food Consumption	-	1.04 (1.00–1.08)	1.03 (0.99–1.08)
Fitness	-	-	0.55 (0.47–0.64)

*Logistic Regression modelled with No MetS group as referent (Model 1: n = 906 Model 2: n = 783, Model 3: n = 777); Sample size for each model varies according to variables included; p<0.05; Abbreviations: PA, Physical Activity; MetS, Metabolic Syndrome;

† - Gender (Female vs. Male), Ethnicity (Black vs. White), Family History (Yes vs. No), Education (Less than High School, High School, College/University, Graduate Degree), Smoking (Non-, Former, Current Smoker), Drinking (Non-, Moderate, Heavy Drinker), Total PA Score (Inactive, Moderately Active, Active), Currently Dieting (Yes vs. No), Fast Food Consumption (Frequency of Visit/Week); Aerobic Fitness (Unfit, Moderately Fit, Fit).

### Non-Modifiable Risk Factors

Results of Model 3 reveal that females and those with a family history of CHD were 37% and 61% more likely to be in the At-Risk group, respectively. Gender was not a significant predictor of group membership upon inclusion of modifiable risk factors.

### Modifiable Risk Factors

Of the six modifiable risk factors assessed in Model 2, baseline education, PA, fast food consumption and engagement in weight reducing diets were all significantly related to the pattern of MetS development, with poorer health behaviours within the At-Risk group. Individuals with low educational attainment and who were dieting at baseline were 28% and 92% more likely to be in the At-Risk group, respectively. Regular fast food consumption was also more common in the At-Risk group (OR: 1.04, 95% CI: 1.00–1.18), an effect that was independent of current dieting practice. Lastly, higher levels of PA were associated with lower likelihood of At-Risk (0.88, 0.79–0.98) group membership; however, both PA and fast food consumption were no longer a significant predictor of group membership upon inclusion of cardiorespiratory fitness, whereas increasing aerobic fitness was inversely associated with At-Risk group status (0.55, 0.47–0.64) in Model 3.

## Discussion

The present study aimed to identify distinct groups of longitudinal MetS development and to develop a character profile of group membership. To our knowledge, this is the first study to use exploratory group-based modelling of longitudinal data to identify heterogeneity of long-term MetS development among a cohort of apparently healthy young adults. By way of this approach, we were able to identify intermediate groups of MetS development that may not be captured in conventional analytic approaches.

Over 20 years of follow-up, 4 distinct trajectories for MetS development were found. It is well known that adolescence and young adulthood is a critical period for the initiation of CVD [20], [21] and that MetS prevalence increases across the lifespan [22], [23]. However, given the young age of participants and CARDIA exclusion criteria, it is not surprising that the majority of participants had a low Framingham Risk Score and low likelihood of MetS development after 20 years. Despite this, these results reveal that the majority of CARDIA participants are actively developing MetS components, while remaining in a pre-disease state. More research is necessary to determine the extent to which the four trajectories may represent different clusters of MetS components and to assess their influence on future health risk [6].

### Character Profiles of High-Risk Groups

Characterization of At-Risk groups (i.e. Low, Moderate and High MetS trajectory groups) that are steadily developing MetS components has important clinical implications for therapeutic intervention. First, the overall prevalence of MetS among Non-Hispanic black individuals in the U.S. is lower than that of both Hispanics and Non-Hispanic whites [22]. From a physiological standpoint, black individuals consistently demonstrate a healthier lipid profile (higher HDL-C, lower TG, and lower LDL-C) but greater insulin resistance, hypertension, and obesity [24]–[27]. Although descriptive and logistic regression results revealed that black participants were more likely to belong to the At-Risk group, the possibility of residual confounding cannot be excluded.

Observed ethnic differences may be further complicated by gender differences within each race/ethnicity. While extensive examination of ethnic-gender stratified models were limited due to the small sample size, preliminary analyses identified four distinct trajectories among black women, and two groups among white women. The additional trajectories observed among black females suggest a high-risk, but heterogeneous group with rapid development of MetS components. Amongst this study's cohort, black females reported a higher baseline prevalence of obesity (20%) than their white counterparts (6%) (results not presented). By contrast, both black and white males had similar trajectory plots with three distinct groups each. However, given the small sample size and exploratory nature of these analyses, interpretation of stratified models must be done with caution.

Current ATP III-R treatment guidelines recommend lifestyle modification (diet, physical activity, and weight management) as the first line of treatment for metabolic dysfunction [11], with LDL-C level as the primary target for CHD risk reduction. In the current study, increasing MetS trajectory corresponded with greater global CVD risk, and more aggressive LDL-C goals (Table 1). At year 20, 7.1% of participants were found to be within the highest risk ATP III-R group, which translates into over 13% of CARDIA participants being eligible for either therapeutic lifestyle change (7.9%) or lipid-lowering therapy (5.7%). While the proportion of participants requiring clinical intervention remains low, these data provide important insight into the natural history of CVD progression through the period of young adulthood. A broader understanding of the factors that accelerate the development of cardiovascular risk are likely to provide further perspective on the primary prevention of MetS and (other key intermediate states of risk) that may otherwise require long-term pharmacotherapy.

Key behavioural and lifestyle factors that must be considered in future analyses include diet (e.g. average caloric intake, macronutrient composition of diet, dietary restriction, and fast food consumption), PA and fitness, and weight management. In the present study, self-reported dieting was predictive of group membership. Specifically, High MetS members were 50% more likely to report current dieting. It can therefore be inferred that at-risk participants may have recognized their need to reduce their weight and manage their diet; however, from these results, it is also clear that as a group their dieting efforts were unsuccessful in light of the general increase in BMI and WC across all follow-ups. This is consistent with a study by Field et al. [28], whereby female dieters were more likely to become obese than their non-dieting peers over a 7-year span, regardless of their baseline BMI. Self-reported dieting may therefore reflect a spectrum of weight loss approaches stemming from short-term, unsustainable approaches to weight loss that could contribute to reflexive overeating that may negate any positive effects of restricted intake [28]. When not accounting for cardiorespiratory fitness, fast food consumption frequency was also identified as an independent predictor of At-Risk group membership, accounting for 4% greater likelihood for those who consume more than two meals per week at a fast food restaurant. In a previous analysis of CARDIA, baseline fast food frequency was associated with increasing body weight and insulin resistance, independent of other dietary factors such as total energy and saturated fatty acid intake [29].

Although the impact of PA on body mass, blood pressure, plasma glucose, and regulation of blood lipid profile has been widely described [30], the magnitude of its contribution to the observed baseline group differences remains unclear. In our study, increased PA levels were inversely associated with High Risk group membership, accounting for 12% reduction in risk with every increase in PA level. However, this relationship did not persist upon inclusion of cardiorespiratory fitness. Similar to other prospective studies [31]–[33], cardiorespiratory fitness attenuated the association between PA and MetS [31]. Although we have shown that higher levels of baseline fitness were associated with a lower likelihood of At-Risk group membership, these results should not be interpreted to diminish the importance of PA participation in the prevention and treatment of MetS, as the environmental contribution to aerobic fitness capacity still exceeds that of genetics [34]. As improvements in cardiorespiratory fitness are largely determined by increased engagement in aerobically-focused physical activity [35], physical activity remains an important therapeutic target for global CHD risk reduction.

### Strengths and Limitations

There are several strengths and limitations of the current study that warrant discussion. First, PROC TRAJ is ideal for evaluating the trajectory of change in MetS over time, with a particular focus on identifying multiple distinct patterns and modelling unobserved variance in data [17]. Since this method assumes that there are clusters of unique developmental trajectories, we are able to forgo the assumptions made by hierarchical and latent curve modelling, wherein the growth or decline of a population is assumed to be in a multivariate normal distribution [36]. Nevertheless, these analyses are exploratory and must be interpreted with caution. In particular, these analysis are based on *a priori* expectations regarding the number, shape, and size of trajectory groups [37], which in the absence of pre-existing literature on individual variation in MetS development, were based on BIC, posterior probabilities, and univariate analysis. While our results are aimed to identify distinct groups, the trajectories presented here are approximations as opposed to exact entities. Second, follow-up examinations from years 2 and 5 were omitted from the current analyses due to lack of glucose measurements and may have resulted in a different trajectory pattern had they been included. Third, as this analysis classified MetS cases on the basis of the Harmonized definition, alternative operational criterions may offer differing patterns of development. Distinct baseline group differences in WC, TG, and HDL-C observed here are most reflective of constructs such as the hypertriglyceridemic waist [38] or the previous International Diabetes Federation definition [39] in which abdominal obesity was a necessary component. Preliminary analyses suggest that although the same high-risk individuals would be identified, the mandatory inclusion of abdominal obesity led to models that did not capture intermediate groups of MetS development (Low and Moderate MetS) we report here. Although cause and effect cannot be inferred, when taken together, these results reinforce the role of abdominal obesity as a key factor in MetS development. Finally, it is unclear whether the patterns observed are generalizable to the broader U.S. population, and the extent to which recall and self-report bias within the survey portion of the study could have influenced the observed associations with modifiable and non-modifiable risk factors.

### Conclusions

Although these analyses are theoretical in nature, the finding of distinct MetS trajectories provides insight into critical periods for lifestyle intervention, risk factor management, and the required aggressiveness of treatment within At-risk subgroups. Most importantly, these analyses not only capture the way in which various groups develop MetS, but also identify character profiles of individuals who are steadily developing MetS, but remain clinically unidentifiable in terms of frank diabetes, hypertension, and CVD. Additional research in large multi-ethnic samples is necessary to understand the importance of ethnic and gender differences in these patterns, and to assess the clinical implication of intermediate MetS development groups with respect to overall cardiometabolic risk.

## Supporting Information

Table S1 **Bayesian Information Criterion (BIC) Model Selection.**
(DOCX)

Table S2 **Posterior Probability by Trajectory Group.**
(DOCX)
