# Supplementary material for: Trajectories of Metabolic Syndrome Development in Young Adults
Source: PLoS One. 2014 Nov 4;9(11):e111647. doi: 10.1371/journal.pone.0111647 (PMC4219745; doi:10.1371/journal.pone.0111647)
Supplement: Table S1 — Bayesian Information Criterion (BIC) Model Selection. (DOCX) [file pone.0111647.s001.docx]

Table S1. Bayesian Information Criterion (BIC) Model Selection.

|  |  | |  |  |  |
| --- | --- | --- | --- | --- | --- |
|  | Probability of Metabolic Syndrome | | | Number of Metabolic Syndrome Components | |
| Number of Groups | BIC (n=16948) | BIC (n=3804) | | BIC (n=17316) | BIC (n=3804) |
| 1 | -4954.25 | -4951.26 | | -24045.32 | -2443.05 |
| 2 | **-4237.50** | **-4233.76** | | -210531.53 | -21529.23 |
| 3 | - | - | | -21050.83 | -21042.50 |
| 4 | - | - | | **20912.72** | **-20902.11** |
| 5 | - | - | | - | - |

MetS-Metabolic Syndrome
