# Supplementary material for: Trajectories of Metabolic Syndrome Development in Young Adults
Source: PLoS One. 2014 Nov 4;9(11):e111647. doi: 10.1371/journal.pone.0111647 (PMC4219745; doi:10.1371/journal.pone.0111647)
Supplement: Table S2 — Posterior Probability by Trajectory Group. (DOCX) [file pone.0111647.s002.docx]

**Table S2.** Posterior Probability by Trajectory Group

|  |  | Groups | | | | | | | |
| --- | --- | --- | --- | --- | --- | --- | --- | --- | --- |
| Group | n (%) | No | | Low | | Moderate | | High | |
|  |  | Mean | Range | Mean | Range | Mean | Range | Mean | Range |
| No | 906 (23.8) | **0.85** | **0.46-0.99** | 0.15 | 0.01-0.50 | 0 | 0.00-0.34 | 0 | 0.00-0.00 |
| Low | 1273 (33.5) | 0.08 | 0.00-0.50 | **0.82** | **0.48-0.99** | 0.1 | 0.00-0.50 | 0 | 0.00-0.01 |
| Moderate | 1342 (35.3) | 0 | 0.00-0.32 | 0.01 | 0.00-0.50 | **0.83** | **0.36-0.99** | 0.06 | 0.00-0.50 |
| High | 283 (7.4) | 0 | 0.00-0.00 | 0 | 0.00-0.00 | 0.16 | 0.00-0.50 | **0.84** | **0.51-1.00** |
